# Eicosanoids in the Innate Immune Response: TLR and Non-TLR Routes

**DOI:** 10.1155/2010/201929

**Published:** 2010-06-15

**Authors:** Yolanda Alvarez, Isela Valera, Cristina Municio, Etzel Hugo, Francisco Padrón, Lydia Blanco, Mario Rodríguez, Nieves Fernández, Mariano Sánchez Crespo

**Affiliations:** ^1^Instituto de Biología y Genética Molecular, Consejo Superior de Investigaciones Científicas, 47003-Valladolid, Spain; ^2^Centro de Hemoterapia y Hemodonación de Castilla y León, 47007-Valladolid, Spain

## Abstract

The variable array of pattern receptor expression in different cells of the innate immune system explains the induction of distinct patterns of arachidonic acid (AA) metabolism. Peptidoglycan and mannan were strong stimuli in neutrophils, whereas the fungal extract zymosan was the most potent stimulus in monocyte-derived dendritic cells since it induced the production of PGE_2_, PGD_2_, and several cytokines including a robust IL-10 response. Zymosan activated *κ*B-binding activity, but inhibition of NF-*κ*B was associated with enhanced IL-10 production. In contrast, treatments acting on CREB (CRE binding protein), including PGE_2_, showed a direct correlation between CREB activation and IL-10 production. Therefore, in dendritic cells zymosan induces *il10* transcription by a CRE-dependent mechanism that involves autocrine secretion of PGE_2_, thus unraveling a functional cooperation between eicosanoid production and cytokine production.

## 1. Polymorphonuclear Leukocytes and Eicosanoid Production

Myeloid cells are unique cell types as regards their content of high amounts of esterified arachidonic acid (AA) and the enzymes necessary to metabolize free AA into different products via cyclooxygenase and lipoxygenase pathways. However, unlike mast cell, which readily respond to cross-linking Fc*ε*RI receptors, and platelets, which release AA in response to classical aggregating agents, less information is available about the physiologically relevant stimuli in polymorphonuclear leukocytes (PMN) and macrophages, since most experiments have been carried out either with xenobiotics or combining chemoattractants with chemicals and priming agents. An example of the first setting has been the use of calcium ionophores. An example of the second situation has been the use of formylated peptides in combination with cytochalasin B and thapsigargin, which extend the time span of calcium transients and allow the occurrence of Ca^2+^ dependent events such as translocation of the cytosolic phospholipase A_2_ (cPLA_2_) from the cytosol to lipid bilayers [1–4]. However, this scenario has suddenly changed with the emergence of new views on the function of the immune system based on the recognition of microbial patterns.

### 1.1. Polymorphonuclear Leukocytes Release Arachidonic Acid in Response to Ligands of Pattern Recognition Receptors

**Figure 1:**
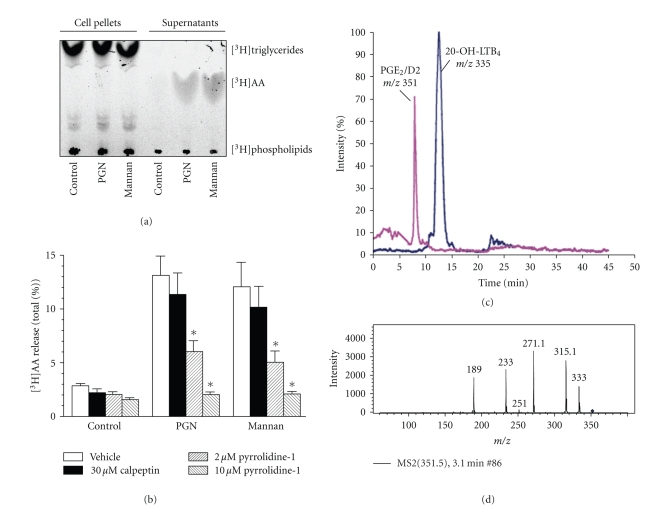
Distribution of [^3^H]AA label in the different lipid fractions in PMN and supernatants. PMN at a concentration of 10^7^ cells/ml were labelled with 0.2 *μ*Ci of [^3^H]AA and stimulated for 1 hour in the presence of 10 *μ*g/ml PGN or 25 mg/ml mannan, or left untreated. PMN and supernatants were subjected separately to extraction in chloroform/methanol (1 : 2, v/v), according to the Bligh and Dyer procedure. The lipids extracted into the chloroform layer were dried under N_2_ stream and developed by thin layer chromatography on silica gel plates in the system n-hexane/diethyl ether/acetic acid (70 : 30 : 1, v/v). The radioactivity distributed in the different lipid fractions was quantitated using K/tritium imaging screens. The migration of the standards is indicated (a). Cells were incubated with calpeptin and pyrrolidine-1 prior to the addition of the stimuli and the [^3^H]AA released into the cell supernatants assayed. Data represent mean ± SEM of three experiments in duplicate. *P < 0.05. Reproduced with permission [10] (b). Chromatogram of a supernatant of human PMN stimulated with mannan showing the retention times of PGE_2_/PGD_2_ and 20-OH-LTB_4_. Both compounds have been identified by their mass spectra (c). Fragmentation spectra in the negative ion model using MRM (multiple reaction monitoring) of the specific transitions *m/z* 335–195 for LTB_4_, *m/z* 351–195 for 20-OH-LTB_4_, *m/z* 303–205 for arachidonic acid, *m/z* 351–189 for PGE_2_/PGD_2_ are shown in (d).

PMN are the first blood cell type able to migrate into tissues following microbial invasion. PMN respond to a large set of stimuli, including inflammatory mediators and microbial products. This group of stimuli is most relevant, since microorganisms have unique molecules, termed pathogen-associated molecular patterns (PAMPs), which are recognized through pattern recognition receptors (PRRs) by the host innate immune system. The Toll-like receptor family (for review, see [5, 6]) and nucleotide-binding oligomerization domain family proteins (NOD) (for review, see [7, 8]) are representative of what Janeway first called PRR [9]. C-lectin type receptors are also PRR that may interact with structural signatures expressed in microorganisms. Experiments in human PMN using as stimuli a set of PAMP signatures including the mannose polymer mannan and peptidoglycan (PGN), a polymer of sugars and amino acids that forms a mesh-like layer outside the plasma membrane of bacteria, showed a robust release of [^3^H]AA (Figures 1(a) and 1(b)) and the production of leukotriene (LT) B_4_ and prostaglandin (PG) E_2_ (Figures 1(c) and 1(d)). The release of [^3^H]AA observed under these conditions was not blunted by calpeptin, an inhibitor of the formation of microvesicles, but was inhibited by treatment with the cPLA_2_ inhibitor pyrrolidine-1. The released product was identified as genuine unesterified AA by thin layer chromatography analysis, since the radioactivity detected in the supernatants showed a *R*_*F*_ distinct from that associated with [^3^H]triglycerides and [^3^H]phospholipids, which were only observed associated with the cell pellets. The release of AA obtained under these conditions was comparable to that elicited by the so far considered reference stimuli such as the formylated peptide combined with thapsigargin or cytochalasin B, and complement-coated zymosan particles. In sharp contrast, stimuli mimicking other bacterial PAMP, that is, lipoteichoic acid, bacterial lipopolysaccharide (LPS), muramyldipeptide (MDP), and the TLR2 agonist Pam_3_CSK4 did not induce AA release [10]. 

The effect of PGN was observed with PGN from both *S. aureus* and *B. subtilis*, thus indicating that PGN displaying the structural features of both Gram + and Gram − bacteria are equally active. Preincubation of PMN with anti-TLR2 mAb prior to the addition of PGN did not inhibit AA release, thus suggesting that TLR2 receptors are not involved in the response to PGN. Consistent with this result, Barrett et al. [11] reported TLR2-independent cysteinyl-LT release from mouse bone marrow dendritic cells stimulated with PGN, since the response was intact in TLR2^−/−^ mice. The assignment of the biological effect of PGN to definite PRR has been a matter of debate [12, 13]. Both TLR and NOD receptors have been involved and the controversy stems from the assignment of the biological properties to either the PGN polymer or the block elements MDP and D-glutamic acid-*meso* diaminopimelic acid. Molecular weight fractionation of *S. aureus* PGN showed the association of AA-releasing activity with fractions of molecular weight >30 kDa, whereas no activity was detected in the <30 kDa ultrafiltrate, which is consistent with the *M*_*r*_ of soluble PGN. The biological significance of the lipid mediators release by PMN in response to TLR ligands was recently underscored in an *in vitro* model of migration through endothelial cell monolayers. In this system, PMN migration was inhibited by LTB_4_ receptor antagonist and platelet-activating factor (PAF) receptor antagonists and was associated with the production of these mediators [14].

### 1.2. Mechanism of Cyclooxygenase-2 Expression Induction in Human PMN

Current understanding of PMN biology has been modified by recent findings indicating that the life span of PMN can be prolonged by proinflammatory agonists [15], and also by the depiction of mechanisms of translational control of the expression of specific proteins that endow the PMN with the potential for rapid protein synthesis from constitutive mRNA without requiring new transcript generation [16–18]. The possibility that this mechanism could be operative in PAMP-dependent responses and might influence AA metabolism through the expression of COX-2, was a challenging hypothesis.

Since PGE_2_ is a major product resulting from AA in the PMN (Figures 1(c) and 1(d)) that can be produced both by COX-1, the constitutive isoform of cyclooxygenase, and COX-2, the inducible isoform, the effect of a set of PAMP signatures on the expression of COX-2 was addressed. Unexpectedly, preformed mRNA encoding for COX-2 was detected in resting PMN, whereas COX-2 protein was only detectable after stimulation with either mannan or PGN [19]. COX-1 protein showed the same level of expression in the absence and presence of several stimuli, but well below the level detected in platelets, which are the archetypal source of COX-1. Pam_3_CSK4 showed a less robust effect and lipoteichoic acid, an agonist of TLR2/TLR6 heterodimers, did not elicit COX-2 protein induction. MDP, which is the archetypal ligand for NOD2, also failed to induce COX-2 expression. Since interaction between NOD2 and specific TLR pathways has been reported as a mechanism of cooperation in the innate immune response that lead to the synergistic activation of host cells [20–22], the effect of the combined addition of both *S. aureus* PGN and MDP was assessed. This combination of agonists did not modify the effect elicited by PGN alone. The induction of COX-2 protein by PGN was observed as soon as 30 minutes after addition of the stimulus and remained almost unchanged from 1 to 18 hours. A similar trend was observed for both C3bi-coated zymosan and mannan, although a decreasing tendency was observed around 18 hours in response to these ligands. These results indicate that PGN contains a structural signature not acting on NOD2 nor mimicked by lipoteichoic acid and Pam_3_CSK4, which could act via the TLR route in combination with an additional catch-up receptor(s) and/or by an as yet ill-defined TLR2-independent route.

**Figure 2:**
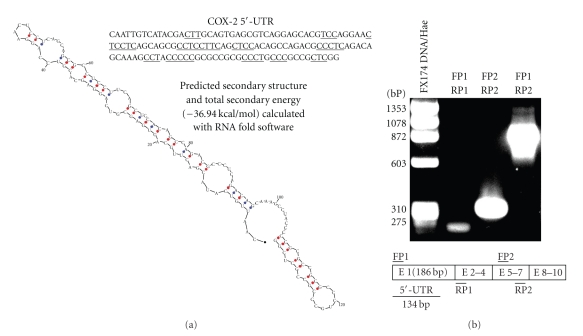
Sequence of the 5′-UTR of COX-2. The sequence and the predicted secondary structure of the 5′-UTR of COX-2 calculated using RNAfold software are shown. Polypyrimidine tracts are underlined in the sequence (a). Agarose gel electrophoresis of PCR reactions in RNA obtained from resting PMN using different combinations of primers (b). Stick diagrams show the location of primers used for PCR reactions, and the lanes are marked according to the combination of primers selected to carry out the reactions. FP indicates forward primer, RP indicates reverse primer. Reproduced with permission [19].

Since PMN are terminally differentiated cells that contain regulators of transcriptional control and show signal-dependent activation of mRNA translation [17, 18], the hypothesis that COX-2 mRNA could be one of those mRNA controlled in the same manner was put forward. Contrary to this view, one could argue that the calculation of the predicted secondary structure energy of the 5′-untraslated region (UTR) of COX-2 mRNA is −36.94 kca/mol, as judged from the application of RNAfold software [23, 24] to sequences available in data banks (Figure 2(a)). This value is lower than that usually associated with transcriptional regulation (−50 kcal/mol); however, it fits well with those reported for many transcripts detected using cDNA library arrays which are regulated at the transcriptional level in human monocytes adherent to P-selectin [25]. Moreover, the presence of four tracts of 5–8 consecutive pyrimidine bases is an additional feature strongly suggesting the possibility of translational control by mammalian target of rapamycin (mTOR). The presence of the 5′-UTR in COX-2 transcripts in human PMN was confirmed by RT-PCR with a set of primers spanning the first 20 nucleotides of exon 1 and exon 2 of COX-2, which gave similar results to PCR reactions using the primers selected from exons 5 and 7 (Figure 2(b)). Preincubation of PMN with 100 nM rapamycin inhibited the induction of COX-2 elicited by complement-coated zymosan, PGN, and mannan, thereby suggesting that the mTOR route is implicated in the translational regulation of COX-2 protein induction. Given that mTOR is integrated in a signalling cascade, the proximal component of which is phosphoinositide-3 kinase (PI3K), the effect of the PI3K inhibitor wortmannin was addressed. A significant inhibition of COX-2 induction was produced by wortmannin as well as by the translation inhibitor cycloheximide. PGN also induced a time-dependent threonine phosphorylation of eIF4E binding protein. This provides further evidence of the involvement of the mTOR route, since the phosphorylation of this translation inhibitor by mTOR disrupts its binding to eIF4E and activates cap-dependent translation [26].

Additional mechanisms of COX-2 mRNA regulation were explored using transcription inhibitors. Actinomycin D did not influence the induction of COX-2 protein elicited by mannan and PGN, whereas it fully inhibited the response to LPS. Since COX-2 mRNA stability in some cell types is regulated at the 3′-UTR, PMN were incubated in the presence and absence of 1 *μ*g/ml actinomycin D for 30 minutes before the addition of PGN to address the half-life of COX-2 mRNA. In the absence of actinomycin D, 53 ± 7% of the starting COX-2 mRNA was detected in control cells versus 70 ± 9% (mean ± SD, *n* = 3) in cells treated with PGN for 2 hours after addition of the stimulus. Actinomycin D treatment induced a further drop of the remaining mRNA in vehicle treated cells, whereas this additional drop was hardly observed in PGN-treated PMN. Further assessment of transcriptional regulation of COX-2 expression was carried out by looking at the effect of 2-hydroxy-4-trifluoromethylbenzoic acid, an inhibitor of both NF-*κ*B and NF-AT [27, 28], which is a useful tool to address in a single step transcriptional regulation since both transcription factors have been involved in COX-2 regulation in different cell types [29, 30]. Hydroxy-4-trifluoromethylbenzoic acid lacked a significant effect on COX-2 protein expression in response to all of the stimuli tested. Taken collectively, these data suggest that transcriptional regulation is not the main mechanism whereby COX-2 expression is regulated in human PMN. 

To ascertain whether the above described mechanisms are either a unique property of PMN or are also operative in other myeloid cells, COX-2 protein expression was assayed in monocytes; however, the time course was somewhat different from that observed in PMN, since COX-2 protein steadily increased up to 4–8 hours. Unlike PMN, rapamycin did not influence COX-2 protein expression in monocytes nor in macrophages, whereas actinomycin D significantly blocked COX-2 protein induction expression in response to zymosan, mannan, PGN, and the soluble *β*-glucan laminarin. These results strongly suggest that different mechanisms can be involved in COX-2 regulation in PMN and mononuclear phagocytes.

## 2. The Macrophage and Dendritic Cell System

**Figure 3:**
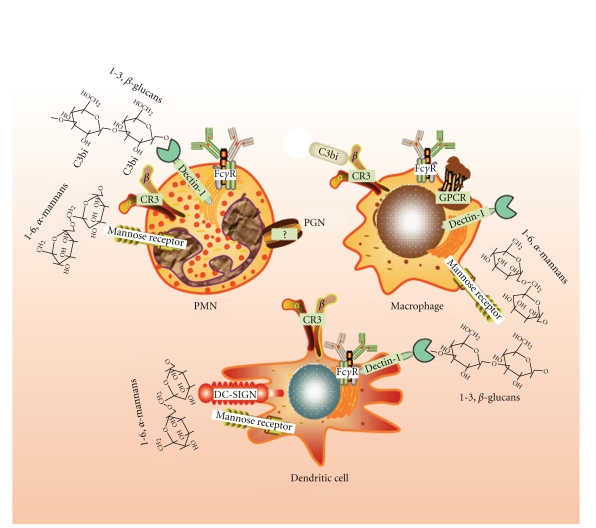
Cells and receptors involved in AA metabolism in the innate immune system. PMN respond to cooperative binding of dectin-1 and CR3, to the cross-linking of Fc*γ*R, to mannose-based PAMP, and to G-protein-coupled receptors (GPCR) in the presence of priming agents. The receptor involved in the response to PGN has not been characterized as yet. Macrophages express a similar array of receptors showing productive binding. In DC the most efficient response is obtained by triggering dectin-1 and DC-SIGN by PAMP containing *β*-glucan and mannose polymers, respectively.

Uptake of phagocytosable particles is strongly dependent on the expression of receptors involved in the recognition of serum proteins displaying opsonic functions such as complement factors and antibodies. This is relevant to the engulfment of fungi and bacteria since these microbes can be coated by the complement factor 3 derived protein C3b and by opsonic IgG class antibodies. The display of receptors on the different cell types including Fc*γ*R receptors, complement receptors, and PRR is a key factor to determine the inflammatory and phagocytic responses and it can widely vary among different cell types (Figure 3). In addition, signals elicited upon binding of receptors by their cognate ligands may be balanced by concomitant signals induced by associated PAMP or from the environment, or even by the expression of cell-specific adaptors [31]. This is particularly relevant to mononuclear phagocytes in view of the different patterns of differentiation they may undergo due to the presence of cytokines and growth factors in the inflammatory milieu.

### 2.1. The Opposing Effect of C3bi-Coating of Immune Complexes and Zymosan Particles on AA Release

AA metabolism was assessed in mononuclear phagocytes stimulated with antigen/antibody immune complexes and zymosan, a cell wall extract of the yeast *Saccharomyces cerevisiae*. Since formation of immune complexes (IC) in fluids containing complement is accompanied by the covalent linkage of C3bi onto the Ab and because C3bi coating of zymosan is known to increase inflammatory responses [32] and AA release in leukocytes [33], experiments were carried out with preformed IC treated with normal human serum to allow the formation of adducts between IgG *γ*-chain and C3b-*α*-chain, a process that has been related to the clearance of IC with a limited inflammatory response. Notably, the AA released by C3bi-IC was significantly lower than that induced by IC containing similar amounts of IgG, thus suggesting that the reaction of IC with C3bi gives rise to an IC lattice showing a distinct ability to interact with signaling receptors. The most likely interpretation of these findings is that the ability of C3bi-IC to interact with complement receptor 3 (CR3) blunts Fc/Fc*γ*R interactions and the attendant AA release associated with Fc*γ*R cross-linking. Treatment of C3bi-IC with anti-C3 IgG, but not with an irrelevant rabbit IgG, allowed the recovery of AA-releasing ability, thus indicating that masking the C3bi moieties with IgG in the C3bi-IC lattice, makes these complexes similar to those formed in the absence of complement [34]. Conversely, removal of the Fc portion of anti-OVA IgG, which preserves the ability of the F(ab′)_2_ fragment to bind covalently C3bi on the Ser-132 of the CH1 domain [35], abrogated AA-releasing activity, thus indicating that Fc-Fc*γ*R interaction is essential for IC-induced AA release and that stimulation through C3bi does not elicit productive binding in this system.

### 2.2. The Role of the Mannose Receptor in Human Monocytes

The mannose receptor (MR), first described by Stahl et al. [36] has been the object of detailed analysis regarding its ability to initiate the uptake of glycosylated molecules with terminal mannose, fucose, or *N*-acetylglucosamine moieties. Its capacity for ligand recognition makes this receptor suitable to phagocytose *Candida albicans, Leishmania donovani,* and* Pneumocytis carinni*, among other microorganisms [37–39]. The MR is the prototypic element of a homonymous family of C-type lectin receptors, which includes the secreted phospholipase A_2_ M-type receptor, the dendritic cell receptor DEC-205, and Endo180/urokinase plasminogen-activated receptor-associated protein. These receptors contain carbohydrate recognition domains, although the chemical structure of the ligands interacting with those domains shows wide differences. The MR is mainly expressed in alveolar macrophages, peritoneal macrophages, and macrophages derived from blood monocytes [40] and seems to play a role in the early immune response against invading pathogens. Although the MR was shown to participate in intracellular signalling leading to target gene expression, the absence of signaling motifs in its intracellular tail makes it necessary the assistance from other receptors in order to trigger any signalling cascade. The MR has been found to exert some effect on the induction of effector T_h17_ cells in mixed leukocytes populations [41] and binding of the mannose polymer mannan to the MR induced a mild expression of COX-2 protein above basal levels, whereas treatment with laminarin, zymosan particles, and preformed IC failed to do so. Notably, monocyte-derived macrophages obtained after two weeks of culture showed a prominent induction of COX-2 protein with concentrations of mannan as low as 0.1 mg/ml, thus suggesting that recognition of mannose-based molecular patterns by macrophages might play a central role in the induction of the innate immune response [42].

### 2.3. AA Metabolism in Monocyte-Derived Dendritic Cells

The main function of dendritic cells (DC) is the detection of pathogens and the initiation of the host response to microbial invasion. So far, few studies have been dedicated to the analysis of the production of AA metabolites [43], in spite of the relevant role of eicosanoids in DC function and the prominent changes in lipid metabolism elicited by M-CSF and IL-4 along the process of monocyte differentiation [44]. In addition, PGE_2_ is required for human DC migration in response to chemokines [45, 46], and consistent with this pivotal function, failure of DC to produce PGE_2_ has been considered a major obstacle for the successful application of DC in therapy [47, 48]. PG biosynthesis involves several steps catalyzed by different enzymes, but it depends primarily on the availability of free AA selectively released from phospholipids by cPLA_2_. COX-2 is involved in the sustained production of prostanoids, the activity of which is necessary for strong Ab response following vaccination [49]. In addition to the COX-2 route for AA metabolism, there are pathways dependent on constitutively expressed 5-lipoxygenase and COX-1, which are triggered shortly after cell activation. As regards 5-lipoxygenase products, deficient extracellular export of LTC_4_ is associated with a decreased migratory response of DC [50], whereas cysteinyl-LT increase IL-10 production by myeloid DC [51]. Recent studies have disclosed lipoxins as a unique class of lipoxygenase interaction metabolites with a strong ability to suppress the production of IL-12 and the function of DC [52].

In keeping with the changes in functional parameters observed upon DC differentiation, AA metabolism in DC showed different patterns in mature and immature DC. Whereas the release of AA elicited by zymosan and other ligands showed no difference between immature and TNF*α*-mature cells, increased expression of COX-2 was only observed in immature dendritic cells. Unlike PMN and monocytes, zymosan particles were the most potent stimulus for AA release, which was observed with concentrations as low as 0.1 mg/ml. In contrast, mannan induced AA release to a lower extent. Unlike the results observed in monocytes, neither C3bi coating nor opsonization with rabbit IgG modified the ability of these stimuli to release AA [53]. This raises important question about the recognition of *β*-glucan particles and the coupled signaling mechanisms in different cell types. In fact, the main receptor involved in *β*-glucan recognition is dectin-1, which is expressed on the cell surface of PMN, monocytes, and DC; however, DC display a unique response to zymosan particles. At first glance, two mechanisms might explain the different responses: (i) expression in some myeloid cell types of an inhibitor, for example, tetraspanin CD37, that restricts dectin-1-CARD9 signaling [31], or (ii) gain of function of DC by differentiation-induced expression of a receptor cooperating with dectin-1. 

Zymosan-induced AA release was inhibited by laminarin, mannan, and anti-dectin-1 and anti-DC-SIGN mAb, specially when the inhibitors were used in combination. These data would suggest cooperation of both dectin-1 and DC-SIGN in zymosan-induced AA release and would agree with the aforementioned hypothesis of the selective expression in DC of a receptor not expressed in other myeloid cell types. To obtain further insight into the type of receptors involved in the recognition of zymosan by DC, the binding of Alexa-Fluor 488 zymosan was studied in the presence of different inhibitors. Mannan, laminarin, anti-DC-SIGN mAb, and anti-dectin-1 mAb blocked zymosan binding, but combination of these inhibitors enhanced binding blockade. Taken together, these data show the existence of a cPLA_2_-dependent route for AA release in DC that is triggered by the binding of zymosan to dectin-1 and DC-SIGN.

### 2.4. Syk Activity Is Involved in AA Release

Protein tyrosine phosphorylation reactions play a central role in cell signaling through both Fc*γ*R and dectin-1 in murine DC [54, 55]. Since these receptors do not possess intrinsic enzymatic activity, their signal transduction pathways must rely on activation of nonreceptor tyrosine kinases. This explains why the Syk/Zap70 family member Syk has been found to be critical for linking receptor engagement to many early downstream events including calcium mobilization and activation of the Ras/mitogen-activated protein kinase pathway. The involvement of Syk in AA release and COX-2 induction in murine macrophages was first reported by Suram et al. [56], who also showed that AA release and LTC_4_ production stimulated by zymosan and *Candida albicans* were TLR2-independent. Studies in human DC were addressed by examining tyrosine phosphorylation of the kinase (a measure of Syk activation) and the effect of Syk inhibitors. Both IC and zymosan induced the phosphorylation of tyrosines in the activation loop of Syk and Syk inhibitors significantly blunted AA release. However, Syk inhibitors only partially affected zymosan-induced cPLA_2_ phosphorylation [53] and the Syk inhibitor piceatannol blunted the release of AA by 96% and 54% in response to IC and zymosan, respectively. R406, a very specific Syk inhibitor, also inhibited completely the response to IC and reduced zymosan-induced AA release by 30%. Zymosan-induced Syk phosphorylation was also inhibited with the addition of laminarin, but not by anti-DC-SIGN mAb. Taken collectively, these results are consistent with the notion that Syk activity is completely necessary for IC-induced AA release, but it is only partially involved in the signalling mechanism whereby zymosan elicits AA release in DC.

### 2.5. DC-SIGN Coimmunoprecipitates with Dectin-1

**Figure 4:**
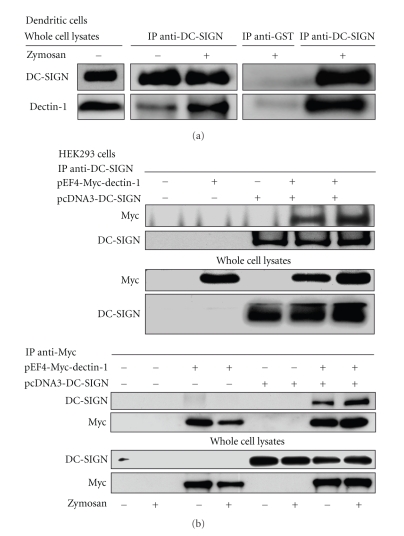
Coimmunoprecipitation of dectin-1 and DC-SIGN. DC were incubated in the presence and absence of 1 mg/ml zymosan for 10 minutes and then the cell lysates were used for immunoprecipitation with either 5 *μ*g anti-DC-SIGN or 5 *μ*g anti-glutathione S-transferase (GST) control mAb and blotting with anti-DC-SIGN, anti-dectin-1, or anti-GST Ab (a). HEK293 cells were transfected with the indicated expression vectors and the cell lysates immunoprecipitated with either 2 *μ*g anti-DC-SIGN or 0.5 *μ*g anti-Myc mAb (b). Upper panels show the expression of the Myc tag and DC-SIGN in the immunoprecipitates. The lower panels show the expression of the proteins in the cell lysates. Cells transfected with pEF4 empty vector were used where no expression vector is indicated. Immunoprecipitations have been conducted with 2 mg protein per condition. The lanes corresponding to cell lysates were loaded with 40 *μ*g protein. Reproduced with permission [53].

**Figure 5:**
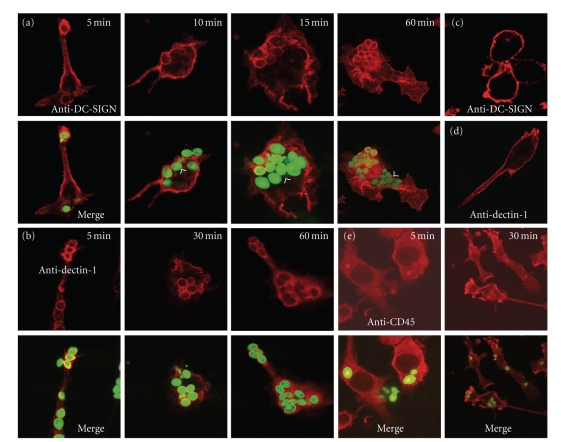
DC-SIGN and dectin-1 cluster around zymosan particles. DC at a concentration of 5 · 10^5^ cells/ml were layered over glass coverslips and incubated with Alexa-Fluor 488 (green) labeled zymosan particles (5 particles per cell) for different times at 37°C. Anti-DC-SIGN mAb and goat anti-mouse IgG Ab labelled with Alexa-Fluor 594 (red) were used for staining DC-SIGN. Note that upon zymosan stimulation, DC-SIGN clusters around zymosan, but not around engulfed particles inside the cell (arrowheads) (a). DC were treated with anti-dectin-1 mAb and goat anti-mouse IgG Ab labelled with Alexa-Fluor 594 (b). Cells incubated in the absence of zymosan particles were stained for DC-SIGN and dectin-1 (c, d). Cells incubated in the presence of zymosan particles were assayed for CD45 staining at the times indicated (e). Reproduced with permission [53].

**Figure 6:**
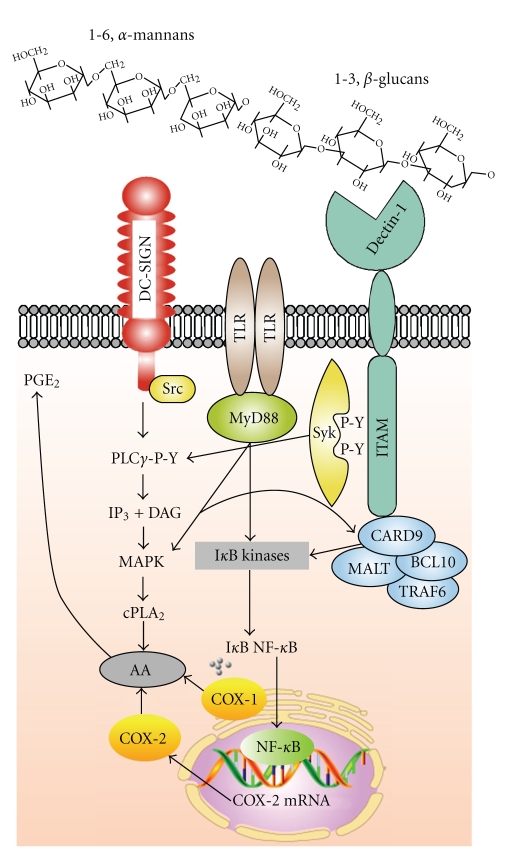
Diagram of AA metabolism in DC stimulated with zymosan particles. The mannan and *β*-glucan components of zymosan are recognized by at least DC-SIGN, TLR2, and dectin-1. This gives rise to a series of signaling events implicating activation of Syk and Src families of tyrosine kinases. Both routes converge to activate phospholipase C*γ* and through the generation of diacylglycerol activate protein kinase C and mitogen-activated protein kinase (MAPK) cascades. Phosphorylation by MAPK and Ca^2+^-driven translocation of cPLA_2_ explain AA release from cell phospholipids. Activation of I*κ*B kinases via MyD88 is a major factor explaining COX-2 induction. The CARD9/MALT1/BCL10/TRAF6 complex is involved in the activation of I*κ*B kinases. DAG, diacylglycerol; IP3, inositol 1,4,5-trisphosphate; PLC*γ*, phospholipase C*γ*; P-Y, phosphotyrosine.

The inhibition of AA release by combinations of laminarin/anti-dectin-1 and anti-DC-SIGN mAb suggested cooperation between DC-SIGN and dectin-1. This was confirmed by showing that dectin-1 coimmunoprecipitated with DC-SIGN, particularly after the stimulation of DC with zymosan (Figure 4). Additional experiments in HEK293 cells transfected with vectors encoding DC-SIGN and Myc-dectin-1 showed a robust coimmunoprecipitation of both C-lectin receptors when immunoprecipitation was carried out with either anti-DC-SIGN mAb or anti-Myc mAb. These results are consistent with a system for zymosan recognition in DC involving the interaction of dectin-1 and DC-SIGN. Studies by confocal microscopy confirmed these findings by showing DC-SIGN clusters in areas of contact with zymosan particles, but not around engulfed particles as judged from the analysis of images taken after 10 minutes, where ingested particles were not surrounded by DC-SIGN staining (Figure 5). This finding agrees with recent reports indicating that DC-SIGN is a mannan-inhibitable zymosan receptor, but does not mediate phagocytosis [57, 58]. In contrast, engulfed zymosan particles were clearly surrounded by dectin-1. Taken collectively, these data would suggest that the differentiation of human monocytes into DC is accompanied by the induction of DC-SIGN, a receptor that cooperates with dectin-1 to elicit an active metabolism of AA. Further support of the role played by changes associated to the process of DC differentiation on AA metabolism is the enhancement of dectin-1-mediated AA release in alveolar macrophages by GM-CSF, a cytokine used to promote DC differentiation [59]. In sharp contrast, rat peritoneal macrophages respond to zymosan particles by promoting the mobilization of both type IIA phospholipase A_2_ and cPLA_2_ into the phagosomes in the absence of growth factors and cytokines [60, 61]. Taking collectively, these findings underscore the importance of environmental factors on the ability of mononuclear phagocytes to regulate the catalytic properties of phospholipases A_2_. A diagram of the signaling routes involved in AA metabolism in DC stimulated with fungal stimuli is shown in Figure 6.

### 2.6. AA Metabolites and the Release of Cytokines from DC

Fungal PAMP acting through dectin-1 and DC-SIGN induce a cytokine response characterized by a high production of IL-10 and IL-23, and a low secretion of IL-12 p70 [55, 62, 63], as compared to the effect on IL-12 p70 production of archetypal TLR4 agonists. This fact may have pathophysiological consequences for the persistence of infection and raises the question of the signaling pathways involved in the predominant IL-10 response. The regulation of IL-10 production has been the subject of intense research in TLR4-dependent models and both transcriptional and posttranscriptional mechanisms have been reported. As regards transcriptional regulation, many transcription factors have been considered as master regulators, namely Stat3 [64–67], Sp1 and Sp3 [68, 69], c-Maf [70], NF-Y [71], NF-*κ*B [72–74], Pbx1b (pre-B cell leukemia transcription factor-1b) [75], c/EBP [76], NFAT [77, 78], and CREB [79, 80]. In addition, posttranscriptional regulation of IL-10 message has also been proposed because of the high number of AU-rich elements in the 3′-UTR of IL-10 mRNA [81] and their binding by the RNA-binding protein tristetraprolin, which destabilizes the message [82]. After addressing the stability of IL-10 mRNA in the presence of actinomycin D, it was concluded that the regulation of IL-10 expression is best explained by transcriptional mechanisms [83]. Computer analysis of human and mouse *il10* promoters was carried out using the MatInspector program and the TRANSFAC database to detect binding sites for transcription factors. In addition, both sequences were aligned with DNA Block Aligner software to define conserved areas, since these regions are more likely to represent functionally relevant elements. Several of the sites detected were previously associated with the transcriptional regulation of *il10*, but there have been some discrepancies regarding their functional relevance and studies with fungus-related stimuli have not been reported. The first approach was to search for the presence of binding activities to the consensus strings of the transcription factors found in the human *il10* promoter. No binding activity to Stat and C/EBP consensus sequences was observed in the nuclear extracts of cells treated with zymosan, whereas binding activity to Stat1 and Stat3 was elicited by IFN-*α*. Constitutive binding activity to Sp sites compatible with both Sp1 and Sp3 was detected, as well as binding activity to CRE consensus sequences. NF-*κ*B is activated by zymosan and has been associated with the regulation of *il10* in mouse macrophages [72–74] and with the regulation of COX-2. Taking into account that the expression of COX-2 parallels IL-10 induction, experiments were conducted using probes containing the *κ*B sites from the human *c* *o* *x*2 promoter that have found to be of functional relevance. Zymosan and LPS were strong activators of NF-*κ*B binding activity to *cox2* sites. The response to zymosan was dose-dependent and binding was competed by the unlabeled sequence. However, as the sequence involved in NF-*κ*B-dependent regulation of *il10* expression in the mouse is not conserved in the human *il10* promoter, the appearance of *κ*B-binding activity in the nuclear extracts upon zymosan challenge is not a proof of the involvement of NF-*κ*B in the regulation of IL-10 expression in human DC. Altogether, the above-mentioned results did not support the involvement of Stat1, Stat3, and c/EBP in the regulation of IL-10 induction and further experiments were conducted focusing on the possible involvement of NF-*κ*B and CREB.

### 2.7. Effect of the Pharmacological Modulation of CREB and NF-*κ*B Activities on IL-10 Production

Since the activity of CREB and NF-*κ*B can be modulated by pharmacological tools, experiments were conducted with 8-Br-cAMP, a cell permeable analogue of cyclic AMP, PGE_2_, and the protein kinase A inhibitor H-89. Elevation of the intracellular levels of cyclic AMP by both PGE_2_ and 8-Br-cAMP had on its own a limited effect on IL-10 production, whereas stimulation with zymosan in the presence of these compounds produced a synergistic increase of IL-10 production. Notably, the opposite effect was observed in the presence of the protein kinase A inhibitor H-89. In contrast with the effect of the chemicals producing inhibition of CREB activity, blockade of NF-*κ*B activity by two structurally unrelated inhibitors was accompanied by an increase of IL-10 production. Since the regulation of CREB activity has been related to calcium/calmodulin kinases and CRE coactivators, the activity of which depends on a sensor of both Ca^2+^ and cyclic AMP levels [84], the Ca^2+^-dependence of IL-10 production was addressed. IL-10 production was blunted by Ca^2+^-chelation. Ionomycin induced a limited production of IL-10, thus suggesting that intracellular Ca^2+^ levels are not the only factor determining IL-10 production. Moreover, low micromolar concentrations of cyclosporin induced a significant decrease of IL-10 production, thus pointing to the involvement of calcineurin in the regulation of IL-10 production. Since E prostanoid receptors type 2 and 4 are involved in the regulation of the intracellular levels of cyclic AMP and zymosan is a strong inducer of COX-2 and PGE_2_ production, the effect of inhibiting COX-1 with the specific inhibitor SC560 and COX-2 with the specific inhibitor NS398 was addressed. The isolated addition of any of those compounds at concentrations of ~1 *μ*M to preserve their selectivity, did not show any significant effect on IL-10 production. Notably, combination thereof produced a significant inhibition, thus suggesting that both COX isoforms might be involved in an autocrine production of PGE_2_ that regulates intracellular cyclic AMP levels and zymosan-induced IL-10 production. Taken together, these results suggest that the polarization of DC cytokine response versus IL-10 production in response to the fungal surrogate zymosan depends on a fine-tuned balance between NF-*κ*B and CREB activity, and that PGE_2_ plays a role in this balance.

### 2.8. The Role of Different Transcription Factors on IL-10 Induction

To address directly the involvement of the distinct transcription factors on IL-10 regulation, chromatin immunoprecipitation (ChIP) assays were conducted using antibodies reactive to P-CREB, CBP, c-Maf, NF-YA, Sp1, and Pbx1. Significant binding of P-CREB to the *il10* promoter was observed in DC stimulated with zymosan, but not in control cells nor in samples treated with an irrelevant antibody. Notably, this was associated with a 64-fold increase of the amount of CBP associated to the *il10* promoter, thus suggesting that zymosan induces both binding of P-CREB to CRE sites and recruitment of the coactivator CBP. ChIP was negative when the PCR reactions were carried out using primers from the *IL12* p35 promoter, which does not contain CRE sites. P-CREB binding was also detected in the *cox2* promoter upon zymosan stimulation, which agrees with the presence of two CRE sites in this promoter and with the functional relevance of these sites in *cox2* transcriptional regulation [85, 86]. Binding of P-CREB and CBP to the promoters was coincidental with the detection of TORC2 (transducer of regulated CREB activity), a CREB coactivator also known as CREB-regulated transcription coactivator (CRTC), in the nuclear extracts. In addition, TORC2 was found to coimmunoprecipitate with P-CREB. 

Expression of the mRNA encoding both the long and the short form of c-Maf was detected in DC, thus agreeing with the reported induction of this factor by LPS and IL-4 in monocytes [70], but binding to the *il10* promoter was not detected by ChIP assays. As regards Sp1 and Sp3, the detection of binding activity in resting cells was not accompanied by binding to the *il10* promoter, which agrees with the notion that this family of transcription factors behaves as a constitutive activator of housekeeping genes and TATA-less genes. Stat3 has been associated with *il10* transcriptional activation, especially in response to ligands of TLR4, which differ from zymosan because of their capacity to activate the Jak/Stat pathway by TRIF (TIR-domain-containing adapter-inducing interferon-*β*)-dependent mechanisms. Stat binding activity and tyrosine-phosphorylated Stat1 were not detected in nuclear extracts from zymosan-stimulated DC, whereas they were induced upon LPS and IFN-*γ* treatment [87]. These results show a major role for CREB in the transcriptional regulation of *il10* in response to the fungal stimulus zymosan.

### 2.9. Langerhans Cells and the T_h17_ Response

The response of DC to fungal glucans is characterized by a high production of IL-23 and the development of a T_h17_ response. This is of interest because T_h17_ cells have been implicated in a number of inflammatory and autoimmune diseases, including multiple sclerosis, inflammatory bowel disease, asthma and psoriasis. So far, only preliminary data have suggested the involvement of lipid mediators in the expansion of T_h17_ cells. The phospholipid mediator PAF is released in response to zymosan in many cell types and is found in increased concentrations in inflammatory lesions. PAF has been shown to induce the production of IL-6 and the development of T_h17_ cells when added at picomolar concentrations to monocyte-derived Langerhans cells and to keratinocytes. Moreover, when Langerhans cells (LC) were pretreated with PAF and then cocultured with anti-CD3- and anti-CD28-activated T cells, the latter developed a T_h17_ phenotype, with a 3-fold increase in the expression of the transcriptional regulator ROR*γ*t and enhanced production of IL-17, IL-21 and IL-22. PAF-induced T_h17_ development was prevented by the PAF receptor antagonist WEB2086 and by neutralizing antibodies to IL-23 and IL-6. It was also dependent on LC-T cell contact as shown in Transwell experiments [88]. These data suggest that a lipid mediator, the biosynthesis of which is associated to the eicosanoid cascade, can stimulate LC to produce IL-6 and IL-23, which, in contact with TCR-activated T cells, can induce their differentiation into T_h17_ cells. This may constitute a previously unknown stimulus for the development and persistence of inflammatory processes that could be amenable to pharmacologic intervention.

## 3. Concluding Remarks

Release of AA and the sequential production of eicosanoid are a blatant outcome of PRR binding by their cognate ligands. The amounts of eicosanoids released under these conditions make PAMP the most potent and physiologically relevant stimuli of AA metabolism in myeloid cells. However, there are a number of significant differences regarding the effect of PRR ligands on the different cell types, even though the same types of receptors might be expressed. This raises relevant questions regarding the distinct signaling routes coupled to the receptors, the role of the concomitant expression of other receptors recognizing the same or other PAMP present on the same ligand, and the effect of positive and negative regulators. Of particular interest is the fact that PGN is the most relevant stimulus in PMN, thus underscoring the important role played by these cells in the pyogenic infections produced by Gram + bacteria. Complement coating of PAMP seems essential for the activation of PMN and monocytes by particles mimicking the fungal cell wall, whereas monocyte-derived DC are a cell type specially endowed to respond to fungal patterns, even in the absence of opsonins. This property depends on the particular set of receptors expressed on their membranes, which cooperate to recognize *β*-glucans and mannose-containing patterns. The signaling network triggered by the combined binding of dectin-1 and DC-SIGN in DC is specially suited for the transcriptional regulation of a pattern of cytokines characterized by a low production of IL-12 p70 and a high production of IL-10. This can be explained by the activation of the transcription factor CREB through a mechanism involving the coactivators CBP and TORC2/CRTC2, and autocrine production of prostaglandin E_2_. These findings emphasize the need of further work to transform these mechanistic data into valuable tools to treat infectious and autoimmune diseases.

## Abbreviations

AA: Arachidonic acid
ChIP: Chromatin immunoprecipitation
DC: Dendritic cells
DC-SIGN: Dendritic cell-specific intercellular adhesion molecule-3-grabbing non-integrin
IC: Immune complexes
MR: Mannose receptor
MDP: Muramyldipeptide
NOD: Nucleotide-binding oligomerization domain family proteins
LC: Langerhans cells
LPS: Lipopolysaccharide
LT: Leukotriene
mTor: Mammalian target of rapamycin
Pam_3_CSK4: Palmitoyl-3-cysteine-serine-lysine-4
PAMP: Pathogen-associated molecular patterns
PG: Prostaglandin
PAF: Platelet-activating factor
PGN: Peptidoglycan
PI3K: Phosphoinositide-3 kinase
PRR: Pattern recognition receptors
TLR: Toll-like receptors
UTR: Untraslated region.
